# Chance Fracture Pattern Presenting in Proximal Junctional Failure

**DOI:** 10.5435/JAAOSGlobal-D-23-00039

**Published:** 2023-08-04

**Authors:** Shea M. Comadoll, Kenneth J. Holton, David W. Polly, Michael W. Schmitz, Jason J. Haselhuhn, Paul Brian O. Soriano, Christopher T. Martin, Kristen E. Jones, Jonathan N. Sembrano

**Affiliations:** From the Department of Orthopedic Surgery (Dr. Comadoll, Dr. Holton, Dr. Polly, Mr. Schmitz, Dr. Haselhuhn, Dr. Soriano, Dr. Martin, and Dr. Sembrano) and the Department of Neurosurgery (Dr. Polly and Dr. Jones), University of Minnesota, Minneapolis, MN.

## Abstract

We present a case series of proximal junctional failure due to a Chance-type fracture.

**Methods:**

This is a retrospective review of patients who developed proximal junctional kyphosis because of Chance-type proximal junctional failure after spinal fusion for adult spinal deformity.

**Results:**

Fifteen patients were identified (4M:11F). The average age was 61.4 years (range, 39 to 77). The mean time to fracture identification was 25.4 days (range, 3 to 65). The average number of levels instrumented was 6.7 (range, 2 to 17). No patients had antecedent trauma before fracture onset. In 67% of cases with a lumbar upper instrumented vertebra (UIV), there was overcorrection of lumbar lordosis (LL) and/or lower LL. The five cases with a lower thoracic UIV had undergone notable correction of preoperative thoracolumbar junction kyphosis. 14 of 15 patients were treated with extension of fusion. Pedicle screws at the fracture level were salvaged by changing to an anatomic trajectory.

**Conclusion:**

Continued pain at 6 to 12 weeks with radiographs showing an increased proximal junctional angle and cephalocaudal pedicle widening at the UIV should raise suspicion for this unique fracture pattern. A CT scan is recommended. Low bone density, LL and/or lower LL overcorrection, and selection of lower thoracic UIV in the setting of notable thoracolumbar junction correction may contribute to fracture risk.

Proximal junctional failure (PJF) is a severe form of rostral junctional pathology that is likely to present with instability, neurologic compromise, and severe pain. In the literature, PJF has an incidence of 1% to 40%; this rate is determined, in part, by the definition used.^1^ While known to be multifactorial, reported risk factors include low bone mineral density (BMD), higher body mass index (BMI), upper instrumented vertebra (UIV) at the thoracolumbar junction (T11-L1), preoperative sagittal imbalance, greater magnitude of sagittal correction, and either deformity undercorrection or overcorrection.^2-4^

Because of the notable effects PJF has on clinical outcomes, it is important for clinicians and radiologists to recognize it on routine postoperative follow-up imaging. A specific cause of PJF, which has been described in several case reports, is a Chance-type fracture at the proximal and distal ends of long fusion constructs.^5,8^ The tension fracture failure pattern was first reported by Chance in 1948. It is a flexion-distraction mechanism of injury, such as in motor vehicle collisions while wearing a lap-type seat belt.^9^ This fracture pattern is AO Spine Class B1; it typically involves a fracture through the pedicles extending into the vertebral body, sometimes to the superior end plate.^10^

We report on a series of 15 patients who present with a specific acute Chance-type PJF/fracture of the UIV that occurred within 3 months of the index surgical procedure. The goal of this report was to describe the radiographic features of this Chance fracture variant, to report how often it is detected or missed by radiology reads, to look at associated BMI and bone density scores, and to look for associated overcorrection.

## Methods

This study was approved by our institutional review board. Fifteen nonconsecutive cases of early PJF with a specific fracture pattern of the UIV were reviewed. All index surgeries were done between September 2013 and February 2020 by fellowship-trained orthopaedic spine surgeons or neurosurgeons in our institution as single or dual-surgeon cases. Intraoperative CT-based (o-arm) computer navigation was used for screw insertion in each case. A check spin done after placement was used to verify acceptable positioning of all screws and that no iatrogenic pedicle fractures occurred.

Patient electronic medical records were reviewed for demographic data including age, sex, smoking status, BMI, and BMD. Patient surgical reports were used to determine fusion levels. Patient radiographs were reviewed and used to measure both preoperative and postoperative radiographic parameters including lumbar lordosis (LL), L4-S1 lordosis (lower lumbar lordosis [LLL]), pelvic incidence, upper segment contour (USC), thoracolumbar sagittal alignment, and proximal junctional angle (PJA). Preoperative CT scans were used to obtain opportunistic bone density (OBMD) measurements.

USC of the instrumented levels was looked at in the following manner. For cases with lumbar UIV (ie, between L1 and L5), the USC was measured from the superior end plate of the UIV to the inferior end plate of the next caudal vertebra (UIV-1). For cases with thoracic UIV, the USC was measured from the superior end plate of the UIV to the inferior end plate of UIV-2.

The change in LL, LLL, and USC measurements from preoperative to postoperative was recorded, and postoperative LL and LLL were compared with the ideal value for each. Ideal values for LL and LLL were calculated using the formulae: (1) Ideal LL (ILL) = 0.5 pelvic incidence + 28^11^ and (2) Ideal LLL (ILLL) = 2/3 of ILL.^12,13^ Overcorrection was assessed by looking at the mismatch between ILL or ILLL and the measured postoperative values. Degrees of correction were calculated by subtracting preoperative from postoperative LL and LLL. Patients were determined to have overcorrection of LL and LLL if they were at least 10° and 5° more than the ideal values, respectively.^14^

PJA was measured from the superior end plate of the vertebral cephalad to the UIV (UIV+1) to the inferior end plate of UIV-1. This was measured in both the preoperative and immediate postoperative preinjury radiographs. If the first postoperative radiographs already showed the injury, then the intraoperative images were used instead.

Preoperative CT scans were analyzed for OBMD testing using CT attenuation (expressed in Hounsfield units [HU]) at L1 and UIV bodies in both the midsagittal plane and axial plane at the pedicle level as described by Anderson et al.^15^

## Results

All 15 fractures leading to PJF occurred within 3 months of surgery and were confirmed on CT. The average patient age was 61.4 years (39 to 77); average BMI was 31.97 (24.09 to 49.07); and 73% (11) were female (Table 1). The average number of levels instrumented was 6.7 (range, 2 to 17), and 47% (7) received an interbody fusion at the most proximal disk space. The average time between index surgery to identification of fracture was 25.4 days (range, 3 to 65 days). No antecedent trauma was reported by any patient.

**Table 1:** Patient Demographics and Surgical Details

Patient	Age	Sex	BMI	DEXA T-Score	L1 OBMD	OBMD UIV	Levels Instrumented	Interbody Fusions (Cage Angle)	Osteotomies
1	51	F	28.3		164.895	164.895	L1-S1	DLIF L1-2, L2-3, L3-4 (6°) PLIF L4-5, L5-S1^a,b^	SPO L1-2, L2-3
2	51	F	41.1		158.17		L2-Pelvis	TLIF L2-3^b^ TLIF L5-S1 (18°)	PSO L4 PCO L5
3	60	F	40.8	−1.4		118.485	T3-Pelvis	TTIF T4-5 (12°) TLIF T12-L1, L1-2 (18°)^a^ TLIF L5-S1 (10°)^a^	SPO T4-5
4	68	M	28.9				L3-Pelvis	LLIF L3-4, L4-5 (10°) TLIF L5-S1 (15°)	SPO L3-4, L5-S1
5	77	M	29.3		269.23	269.23	L1-Pelvis	TLIF L1-2 (6°)	SPO L1-2 PSO L5 (25°)
6	30	M	35.0	−2.4	136.89	144.54	L4-Pelvis	LLIF L4-5 (15°) TLIF L5-S1 (18°)	SPO L4-5, L5-S1
7	56	F	27.5	−2	125.08	120.655	L2-Pelvis	TLIF L2-3, L3-4 (12°) ALIF L4-5^a,b^ TLIF L5-S1 (18°)	SPO L2-3, L3-4, L5-S1
8	62	F	24.1	−2.6	102.96	96.21	L2-Pelvis	TLIF L4-5, L5-S1 (18°)	PSO L4
9	39	F	30.2		198.425	172.23	L4-Pelvis	ALIF L4-5 (20°) ALIF L5-S1 (15°)	SPO L4-5 SPO and Gill laminectomy L5-S1
10	72	F	32.6	−1	192.685	105.815	T10-Pelvis	TLIF L5-S1 (18°)	SPO L5-S1
11	72	F	22.0	−2.2	124.25	132.93	T11-Pelvis	TLIF L4-5, L5-S1 (18°)	SPO L4-5, L5-S1
12	71	F	27.5		128.11	117.125	T12-Pelvis	DLIF L4-5 (6°) TLIF L5-S1 (18°)	SPO L5-S1
13	77	M	27.2	−1.6	117.125	80.905	T11-Pelvis	TLIF L4-5, L5-S1 (18°)^c^	SPO L4-5, L5-S1
14	56	F	49.1		113.015	127.4	L2-Pelvis	TLIF L4-5, L5-S1 (18°)	SPO L4-5, L5-S1
15	69	F	36.1	−2.4	128.825		T10-Pelvis	LLIF L4-5 (15°) TLIF L5-S1 (12°)	SPO L4-5, L5-S1

BMI = body mass index, DEXA = dual-energy radiograph absorptiometry, DLIF = direct lateral interbody fusion, PLIF = posterior lumbar interbody fusion, LLIF = lateral lumbar interbody fusion, OBMD = opportunistic bone mineral density, PCO = posterior column osteotomy, PSO = pedicle subtraction osteotomy, SPO = Smith-Petersen osteotomy, TLIF = transforaminal lumbar interbody fusion, TTIF = transforaminal thoracic interbody fusion, ALIF = anterior lumbar interbody fusion, UIV = upper instrumented vertebra

a Existing constructs before index surgery.

b Cage angulation unclear.

c Existing left L5-S1 TLIF cage unable to be removed and only right cage revised.

Eight of the 15 patients had preoperative dual-energy radiograph absorptiometry scans. All eight patients had low BMD (T-score < −1.0). Fourteen of the 15 patients had preoperative CT scans on which we measured opportunistic bone density scores at both L1 and the eventual UIV. Average OBMD measurement on preoperative CT scans was 146.36 HU (99.35 to 218.46) at L1 and 134.22 HU (76 to 218.46) at the UIV. One patient did not have a preoperative CT, one patient had prior instrumentation at L1, one had prior instrumentation at their UIV, and one had a CT scan that did not include their UIV, so opportunistic measurements were unable to be obtained.

Patient radiographic measurements are presented in Table 2. Overcorrection of either LL or LLL by at least 10° and 5°, respectively, was seen in most of the patients (9/15), including those who had a lumbar-level UIV (4/9). Four of the five remaining patients with a lumbar-level UIV had LL overcorrection of at least 5°. The five cases with lower thoracic UIV had a mean preoperative thoracolumbar junction kyphosis of 39.6° (range, 17° to 62°) and with subsequent correction of 26° (range, 3° to 35°). Across the cohort of 15 patients, the mean USC change was 11.4°. Twelve (80%) had at least 5° and 9 (60%) had at least 10° lordosis correction in the upper segment next to the UIV.

**Table 2:** Patient Radiographic Measurements

Fracture Level	Patient	Preoperative LL	Ideal LL	Postoperative LL	Preoperative LLL	Ideal LLL	Postoperative LLL	Preoperative USC	Postoperative USC	Preoperative TL	Postoperative TL	Postoperative PJA	PJA After Fracture
T3	3	41	49.5	47	20	33	19	42K	24K	4L	5L	12K	33K
T10	10	33	47	45	47	31	45	23K	26K	62K	27K	9K	27K
T10	15	39	53	65	37	33	39	13K	13K	17K	14K	9K	33K
T11	11	18	53	61	59	35	47	43K	31K	59K	25K	12K	35K
T11	13	12	65	64	26	43	46	25K	9K	33K	9K	2K	22K
T12	12	24	48.5	49	32	32	47	23K	7K	27K	18K	14K	30K
L1	1	33	52.5	67	34	35	40	15K	1K	16K		4K	32K
L1	5	19	54	59	27	36	41	5K	0	9L	0	2K	11K
L2	2	33	59.5	76	10	40	56	9L	25L	21L	6K	0	17K
L2	7	43	52	57	23	35	37	17K	15L	0		5K	13K
L2	8	19	50	58	20	33	34	3L	15L	1K		4L	9K
L2	14	44	64.5	55	43	43	38	8L	14L	8L		3L	13K
L3	4	44	52	50	35	35	43	10L	18L	7K	10K	4K	5K
L4	6	31	45.5	37	22	30	35	21L	21L	4K		1L	9K
L4	9	75	66.5		44	44	49	60L	41L	12L		25L	0K

K = kyphosis, L = lordosis, LL = lumbar lordosis, LLL = lower lumbar lordosis, PJA = proximal junctional angle, TL = thoracolumbar sagittal alignment, USC = upper segment contour

**Figure 1:**
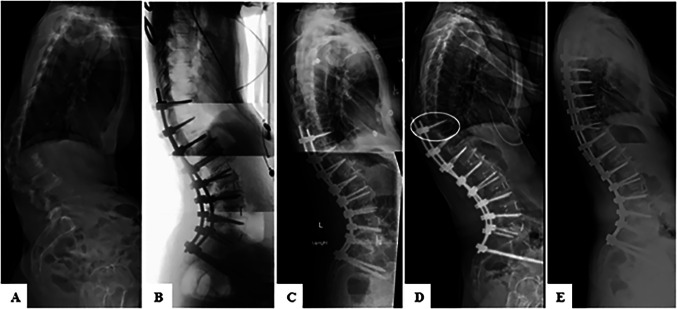
**A**, Lateral preoperative spine radiograph. **B**, Intraoperative radiograph. **C**, First upright radiograph. **D**, First radiograph with fracture present. **E**, Postrevision radiograph with an altered screw trajectory at a prior fracture location.

**Figure 2:**
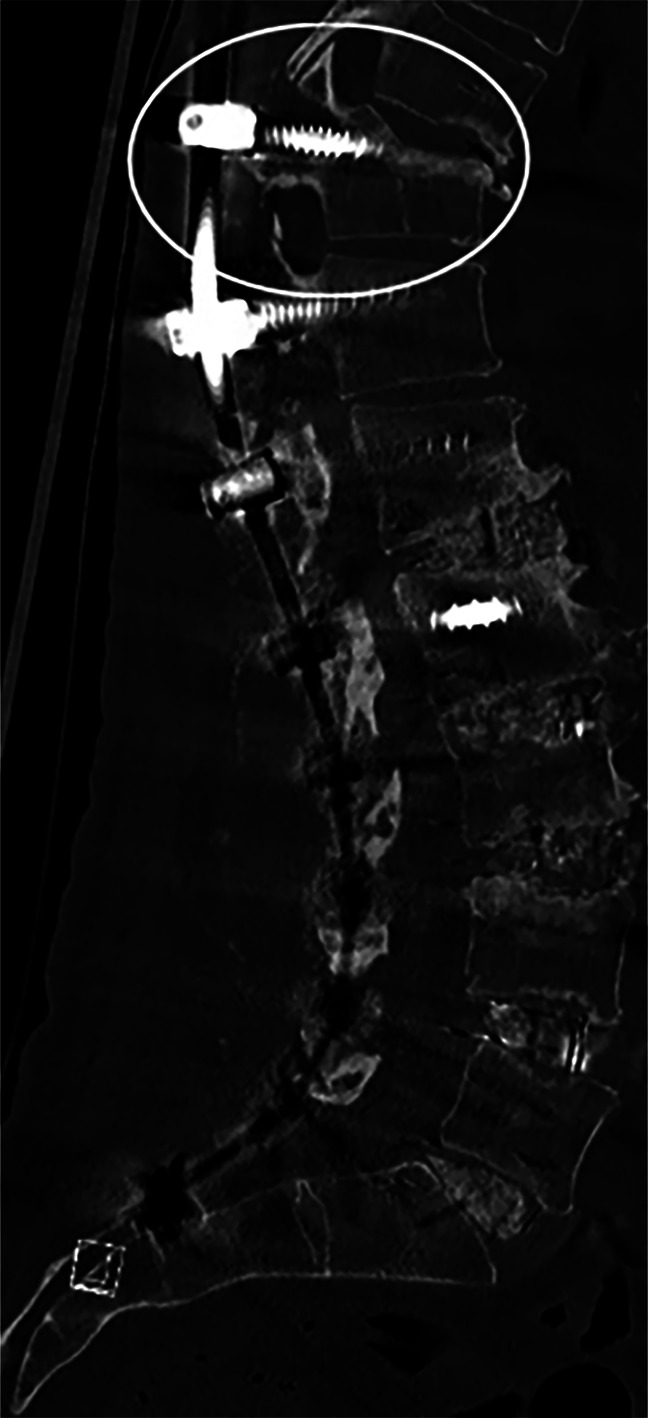
Image showing a computed tomography scan demonstrating a Chance-type fracture pattern.

Postfracture radiographs showed increased PJA and UIV cephalocaudal pedicle widening (Figure 1). The confirmatory CT scans revealed a fracture extending through the long axis of the upper instrumented pedicle, with the cephalad fragment and superior articular process displaced superiorly (Figure 2). In 73% (11/15) of cases, the fracture was not identified on postoperative radiographs by the reading radiologist. Among the patients who underwent CT scan, 46% (6/13) were not identified by the reading radiologist on CT.

Ninety-three percent (14/15) subsequently underwent proximal extension of fusion. Pedicle screws at the fracture level were either removed or salvaged by changing to an anatomic trajectory (Figure 1). One patient was managed conservatively with an anabolic bone agent because of osteoporosis.

In this series, the fractures occurred acutely. Forty-seven percent (7/15) were diagnosed while the patients were still in the hospital, and 93% (14/15) were identified on or before their 6-week radiographs. The one fracture diagnosed at 3 months had evidence of a 12° increase in junctional kyphosis on their 6-week radiographs compared with the immediate postoperative radiographs. In retrospect, the fracture had likely already occurred by that time yet was not confirmed until subsequent imaging was obtained at 3 months postoperatively.

Nine fractures occurred in lumbar levels (L1 to L4). When the sagittal alignment parameters of these cases were analyzed, it revealed that six cases were overcorrected relative either to individualized ILL or ILLL. Five of the remaining six cases with UIV above L1 had fractures in the lower thoracic spine between T10 and T12 while one occurred at T3. The five lower thoracic fractures had an average preoperative thoracolumbar junction kyphosis of 49.5° (range, 17° to 62°) and with subsequent average correction of 26° (range, 3° to 35°).

## Discussion

**Figure 3:**
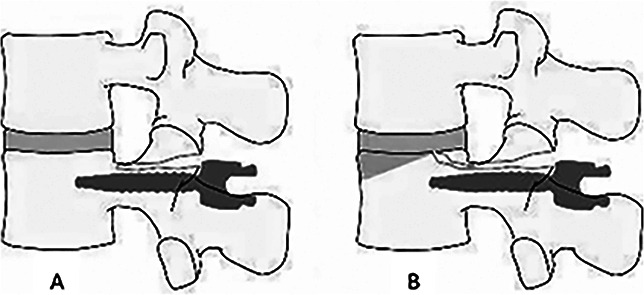
**A**, Diagram depicting a Chance-type fracture pattern with fracture extending through the pedicle. **B**, Diagram showing fracture extending to the superior end plate; shaded area depicts where the vertebral body compression fracture may occur.

This is the first reported case series of the Chance-type fracture pattern of the UIV that present in the early postoperative period (within 3 months) after multilevel spinal fusion down to the sacrum/pelvis. This fracture variant occurs when the pedicles are split along their longitudinal axis into cranial and caudal fragments. Once within the body, the fracture lines exit through the superior end plate and into the disk instead of continuing all the way through to the anterior cortex (Figure 3, A). There also may or may not be a recognizable vertebral body compression fracture (Figure 3, B). The main characteristics appreciated on lateral radiographs include a marked increase in proximal junctional kyphosis (PJK), cephalocaudal widening of the UIV pedicles, and anterior wedge deformity (when compression fracture is present).

We are unable to classify these Chance-type fractures cleanly into the Hart-International Spine Study Group Proximal Junctional Failure Severity Scale because we did not report on neurologic deficit, axial pain, or instrumentation issues.^16^ Using the classification system described by Yagi et al,^17^ all patients would be described as having failure at the implant/bone interface, which would make them Type 3. In this study, three would not be able to be classified because of increase <10 of the PJA, two would be 3A, four 3B, and six 3C.^17^

The average number of levels instrumented was 6.7 (range, 3 to 17), and the location of the UIV where the fractures occurred varied from patient to patient. These findings suggest that the fractures described earlier could occur with varying lengths ranging from short to long constructs. Notably, one patient developed this fracture at the upper thoracic spine while most fractures were noted from T10 and lower levels.

In hindsight, 5 of 6 patients with a UIV above L1 sustained a subsequent lower thoracic fracture (T10-T12) and may have benefited from carrying the fixation higher to the upper thoracic spine. In addition, none of these patients had proximal tethers. It is unknown whether proximal tethers or adjuncts including hooks or cement may have helped avoid this failure pattern.

USC correction was also examined as a potential risk factor. Twelve (80%) had at least 5° and 9 (60%) had at least 10° lordosis correction in the upper segment alone, right next to the UIV. The mean USC change was 11.4°. This suggests that leaving the upper segment neutral relative to preoperative alignment and focusing sagittal correction at lower levels may help reduce PJF risk. While we are not aware of any previous studies that specifically looked at upper segmental contour change next to the UIV as a potential risk factor in the development of PJK/failure, many experts now anecdotally recommend contouring the rods into a gentle kyphosis at the proximal end of the construct.^18^

Low bone density was present in all eight patients with available dual-energy radiograph absorptiometry scans. In addition, opportunistic bone density measurements revealed an average of 146 HU; however, when looking at the data, all but two patients measured below the threshold of 135 HU demonstrated in prior literature as associated with low bone density. This finding is consistent with previous studies showing low bone density as a risk factor of PJF.^19,20^

Based on our review, patients with a Chance-type proximal junctional fracture generally have (1) an increase in PJA (kyphosis), (2) cephalocaudal widening of the UIV pedicles, and (3) possible anterior column compression deformity of the UIV. These findings, in the setting of a patient with persistent or new pain symptoms, should prompt the surgeon to order a CT scan to confirm the diagnosis. If the patient does not have profound/progressive neurologic deficit, a discussion should be had regarding risks of progression and patient goals to determine whether surgical intervention is the best option. In our series, 14 of 15 patients (93%) eventually ended up undergoing revision surgery to extend the spinal fusion and fixation past the fracture level.

An important point to note is that these fractures are not easily detected. The acute occurrence of pain as a symptom of this injury may be masked by expected postoperative pain that occurs after major spine surgery. Multiple factors affect fracture detection. The PJK can be errantly attributed to stress relaxation of the thoracic spine (i.e., it is able to revert to more physiologic kyphosis postoperatively as the lumbar flat back is now corrected). Known barriers to identify this fracture pattern include increased use of low radiation, lower resolution imaging, and shifting emphasis on full-spine or full-body radiographs (thus, more things to look at). The ease at which this fracture pattern can be missed on plain radiographs is clearly demonstrated in this study, in which 11 (73%) were missed by the radiologist on radiographs and six (46%) on CT scans. It should be pointed out that radiologists are blinded to the patient's clinical presentation, whereas the surgeon might have a higher suspicion index if the patient is complaining of notable pain in the top area of the fusion. This only highlights the fact that these findings could be subtle and thus easy to miss. It is important for surgeons following these patients to obtain and personally review upright spine radiographs, especially when patients are reporting continued or new pain symptoms.

A strength of this study is the comprehensive description of the patient population, the surgical intervention done, and the radiographic alignment outcomes in a series of patients where a Chance-type fracture was observed. In addition, we explored several patient and surgical factors to understand what might have contributed to these fractures.

However, there are several limitations to this study. First, this case series presented a heterogeneous group of patients with similar fracture patterns after spine surgery. The number of fused levels ranged from 3 to 17, and patients had varying levels of prior instrumentation. Thus, patients may have different biomechanical forces acting on their construct and the upper instrumented level, leading to fractures. Future studies may explore how these fractures occur in the different spinal regions and construct lengths. Another limitation is the study's case series design; hence, no comparison was made with a group that underwent similar surgery and did not sustain a fracture. Future studies with a control group would allow for statistical analyses and a risk association model with internal validation to be developed to better define risk factors. We also acknowledge that overcorrection of LL and LLL does not completely account for the risk of failure in these patients and that global sagittal malalignment is likely a risk factor of PJK.

## Conclusion

This is a report of 15 cases of Chance-type proximal junctional fractures of the UIV sustained in the early postoperative period among patients who underwent spinal fusion and instrumentation down to the sacrum/pelvis. Common patient features include low BMD, LL overcorrection or nonphysiologic lordosis distribution, and lower thoracic UIV in the presence of preoperative thoracolumbar kyphosis. These fractures are often easily missed in radiology reports; thus, increased awareness of their radiograph features should be emphasized. These include increased junctional kyphosis, cephalocaudal widening of the UIV pedicles, and possible presence of anterior wedge compression deformity. When these features are noted, we recommend that a confirmatory CT scan be obtained, and if fracture is confirmed, options including possible revision surgery are discussed with the patient.
